# The effect of urban morphological characteristics on the spatial variation of PM_2.5_ air quality in downtown Nanjing

**DOI:** 10.1039/d1ea00035g

**Published:** 2021-08-26

**Authors:** Tom V. Kokkonen, Yuning Xie, Pauli Paasonen, Shahzad Gani, Lin Jiang, Bo Wang, Derong Zhou, Wei Qin, Wei Nie, Veli-Matti Kerminen, Tuukka Petäjä, Jianning Sun, Markku Kulmala, Aijun Ding

**Affiliations:** Joint International Research Laboratory of Atmospheric and Earth System Sciences, School of Atmospheric Sciences, Nanjing University Nanjing China tvkokkonen@nju.edu.cn; Nanjing Atmospheric Environment and Green Development Research Institute (NAGR) Nanjing China; Institute for Atmospheric and Earth System Research/Physics, Faculty of Science, University of Helsinki Helsinki Finland; Helsinki Institute of Sustainability Science, University of Helsinki Helsinki Finland; Gulou Environment Protection Department Nanjing China; Jiangsu Environmental Monitoring Center Nanjing China

## Abstract

The effects of the urban morphological characteristics on the spatial variation of near-surface PM_2.5_ air quality were examined. Unlike previous studies, we performed the analyses in real urban environments using continuous observations covering the whole scale of urban densities typically found in cities. We included data from 31 measurement stations divided into 8 different wind sectors with individually defined morphological characteristics leading to highly varying urban characteristics. The urban morphological characteristics explained up to 73% of the variance in normalized PM_2.5_ concentrations in street canyons, indicating that the spatial variation of the near-surface PM_2.5_ air quality was mostly defined by the characteristics studied. The fraction of urban trees nearby the stations was found to be the most important urban morphological characteristic in explaining the PM_2.5_ air quality, followed by the height-normalized roughness length as the second important parameter. An increase in the fraction of trees within 50 m of the stations from 25 percentile to 75 percentile (*i.e.* by the interquartile range, IQR) increased the normalized PM_2.5_ concentration by up to 24% in the street canyons. In open areas, an increase in the trees by the IQR actually decreased the normalized PM_2.5_ by 6% during the pre-COVID period. An increase in the height-normalized roughness length by the IQR increased the normalized PM_2.5_ by 9% in the street canyons. The results obtained in this study can help urban planners to identify the key urban characteristics affecting the near-surface PM_2.5_ air quality and also help researchers to evaluate how representative the existing measurement stations are compared to other parts of the cities.

## Abstract 2

The most important characteristics in terms of the PM_2.5_ concentrations were the fraction of trees and the height-normalized roughness length. The study was performed using continuous observations covering the whole scale of urban densities.

## Graphical abstract

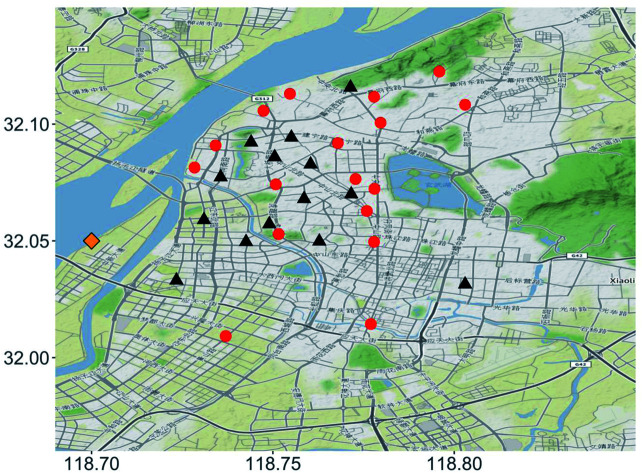

**Environmental significance**

There is substantial spatial variation of near-surface PM_2.5_ pollution in cities. It is crucial to understand the key urban characteristics affecting the local scale air quality so that urban planners could build healthier cities. The effect of nearby trees in street canyons was identified to be the most important urban morphological characteristics defining the near-surface pollutant concentrations and the height normalized roughness length as the second most important. The accumulation of pollutants due to the trees highlights the importance of utilizing scientific knowledge before planting urban trees in street canyons. The results obtained in this paper indicates that the results could be transferred also to other cities even with different emissions scenarios.

## 1. Introduction

The rapid industrialization and urbanization have led to many environmental problems in China.^1–4^ As a consequence of this rapid development, eastern China is one of the most populated areas in the world which suffers also from atmospheric pollution especially in urban areas.^5,6^ The air pollutants have been estimated to cost over 1 million lives annually in China^7^ and fine particulate pollutants have been identified as one of the most harmful air pollutants in terms of human health.^8–10^

The air quality can vary substantially in different parts of the city. This is partly due to differences in local spot emissions,^11,12^ traffic conditions^13–15^ and due to the effect of urban structures on the ventilation conditions in street canyons.^16,17^ Street-level pollutants in urban environment are dispersed and mixed into the upper layers by the wind in street canyons. However, urban morphological characteristics can affect near-surface ventilation conditions and therefore also pollutant concentrations at a pedestrian level.^18,19^ In addition, good ventilation conditions in cities are beneficial not only in terms of air quality but also in terms of restricting the spreading of airborne transmitted diseases like the COVID-19.^20^ Therefore, it is crucial for urban planners to have a better understanding of the effect of urban morphology in order to minimize its negative effects on urban ventilation. However, we are lacking information of the relative importance of the different characteristics.

In recent decades, the effect of urban spatial morphological characteristics has gained more attention in urban climate research. The Local Climate Zones (LCZ) proposed by Stewart and Oke^21^ is considered to be one of the essential studies emphasizing the importance of these characteristics in terms of urban climate. There have been numerous studies in the past examining the effect of urban structures on air quality. However, a majority of these studies are performed for example in wind tunnels,^22^ focusing on idealized street canyons and individual roughness elements,^23^ or by modeling.^24^ Even though modeling studies can give very high-resolution maps of spatial air quality at a street level, they are generally lacking proper evaluation against observations with a high enough spatial coverage.

The effect of trees on air quality in urban environments is still a hot research topic with controversial results. It is generally accepted that urban trees can purify air by absorbing gaseous pollutants and by deposition of particulate matter,^25^ so planting of urban trees have been understood as an effective air pollution mitigation technique in cities.^26,27^ However, based on recent studies, trees can actually deteriorate the air quality on a local scale. Trees are porous and flexible, and can therefore absorb momentum and reduce the turbulent intensity and the velocity of the wind flow, even during the winter when there are no leaves, and therefore trees can deteriorate the air quality locally.^28–32^ Furthermore, certain tree species can release significant amounts of biogenic volatile organic compound (BVOC),^33^ contributing to ozone formation in the atmosphere. However, there are still considerable uncertainties in the net impact of trees on air quality in different urban areas, for example in urban parks and street canyons.

Instead of focusing only on idealized street canyon cases, modelling or few individual sites, we performed this study in a real urban environment using observations in highly varying urban densities. We focused on a few regularly used urban morphological characteristics which can be rather easily defined for any urban areas using commonly available datasets. The characteristics studied are the height normalized roughness length (*z*_0_/*z*_H_), fraction of trees within 50 m radius of the station (*f*^50^_trees_), orthogonal distance to major roads (*D*_road_) and street canyon aspect ratio (*λ*_S_) (see Section 2.4 for details). These characteristics are also assumed to be among the most important ones related to ventilation conditions and therefore influencing the concentrations of near-surface atmospheric pollution.

Our objectives in this paper are (1) to examine the effect of urban morphological characteristics on the near-surface PM_2.5_ mass concentrations, (2) to identify the importance of urban morphological characteristics together and each of the characteristics individually on the spatial variation of the PM_2.5_ mass concentration and (3) to examine the effect of urban morphological characteristics on PM_2.5_ mass concentration during the COVID lockdown under a substantially different emission scenario due to the COVID restrictions. To our knowledge, this is the first study to examine the effect of urban morphological characteristics on air pollution based on continuously observed data covering the whole scale of urban densities typically found in cities and identifying the importance of individual characteristics under different emission scenarios.

## 2. Material and methods

### 2.1 Study site

**Fig. 1:**
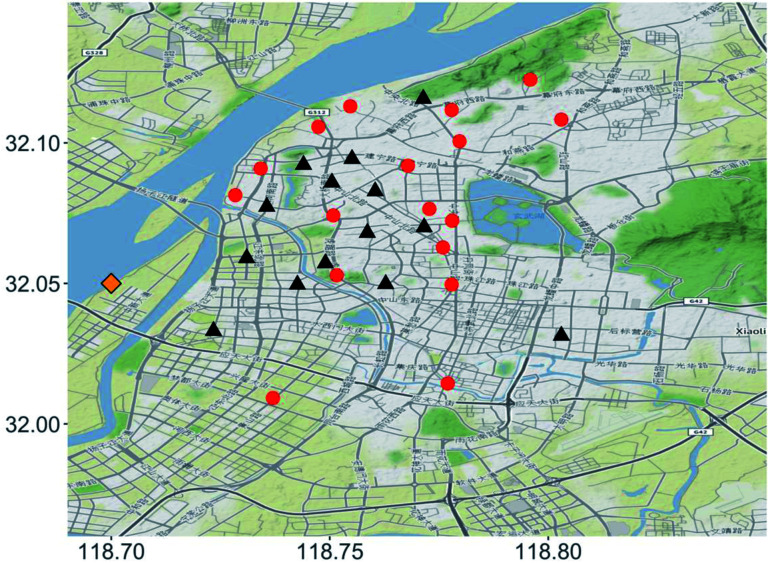
Map showing the locations of the air quality observation stations in street canyons (black triangles) and open areas (red dots) in downtown Nanjing and the meteorological observation station (orange diamond). The background map is obtained from Stamen Design (CC BY 3.0).

In this study, we used continuously measured hourly data from 31 observation stations scattered around the downtown of Nanjing (Fig. 1). A bit more than half of the stations (*N* = 17) are located in rather open areas (*e.g.*, next to a sports field, large parking lot, urban park *etc.*) and the rest of them (*N* = 14) are located in typical street canyons, with buildings directly on both sides of the street.

The study areas are circles with a 500 m radius around each of the stations, since individual or clusters of roughness elements have been shown to affect the downwind wind profiles up to 4–5 times the roughness element height.^34,35^ Therefore, upwind roughness elements in downtown locations can easily affect wind profiles 500 m downwind. The 500 m radius circles are divided into eight different wind sectors (width 45°) and the urban morphological characteristics are determined and the monthly medians are calculated independently for each of the wind sectors, which leads to 248 individual sectors for the analyses.

### 2.2 Instrumentation and data

The hourly PM_2.5_ observations (YSRDAQ-07; Insights Value Technology Co. Ltd) were performed continuously using a recently installed observation network that has been operational since the latter half of 2019. The hourly meteorological data provided by the Jiangsu Meteorological Bureau, including precipitation, wind speed and direction, were measured at the shore of an island located in Yangtze River (Fig. 1). Therefore, it is assumed to be measuring urban background meteorological conditions without major disturbances.

The frontal area and plan area of buildings were determined using a three-dimensional, GIS-based building dataset for the year 2018 extracted from the Gaode Map (https://ditu.amap.com) following the methods described in Zhang *et al.*^36^ The dataset used in this study are covering the Nanjing urban area and were processed with the QGISv3.10 software.

Trees were determined manually from aerial photographs from Bing Virtual Earth processed with QGISv3.10. The fraction of trees was determined for the whole wind sectors, used in the calculation of the aerodynamic roughness parameters (see Section 2.5), and for a 50 m radius around the station in order to examine the effect of nearby trees on PM_2.5_ air quality. The frontal areas of the trees were calculated using the surface area of trees identified from the aerial photographs and the average tree height of 7 m for Nanjing urban trees,^37^ since the information of the height of individual trees was not available.

### 2.3 Data preprocessing and statistical methods

Since urban morphological characteristics are assumed to affect the ventilation of the street canyons by reducing near-surface wind speeds, calm conditions (wind speed <1 m s^−1^) were filtered out from the hourly data. Also, hours with precipitation were filtered out. Hourly PM_2.5_ data were normalized by dividing it with the minimum of all stations, which was assumed to represent the urban background concentration, in order to minimize the effect of meteorological conditions and transported pollutants.

The effect of urban morphological characteristics is assumed to be stronger (or different) for the stations that are located in street canyons than in open areas. Since about half of the stations are located in rather open areas and the other half of them are located in a street canyon (Fig. 1), the analyses were made for the open areas and street canyons separately.

Since the COVID lockdown had substantial effects on the emissions of local pollutants, the analyses were divided into pre-COVID (1 December 2019 to 23 January 2020) and COVID lockdown (24 January 2020 to 29 February 2020) periods.

After the filtering of the data, roughly monthly medians were calculated for each of the eight wind sectors and for each station. For the pre-COVID period, two medians were calculated for the analyses (1 December 2019 to 31 December 2019 and 1 January 2020 to 23 January 2020) and for the COVID lockdown period one median was calculated (24 January 2020 to 29 February 2020). Only the hours with wind coming from the sector were included in the monthly medians for that specific sector. This is also partly the reason why monthly medians were used instead of, for example, daily medians. With daily values, the amount of data used for the averaging might not be sufficient for some of the wind sectors.

Linear regression analysis was used to determine the statistical significance of the relationship between individual urban morphological characteristics and normalized PM_2.5_ concentrations. In addition, the average percentual change of the normalized PM_2.5_ concentration when the characteristics examined increased from 25 percentile to 75 percentile (*i.e.* by the interquartile range, IQR), was calculated using a linear regression fit.

Partial least squares (PLS) regression analysis was used to evaluate the response of the normalized PM_2.5_ concentration on the urban morphological characteristics. The PLS regression method is a multivariate technique combining the features of principal component analysis and multiple regression,^38^ and it is generally assumed to be more statistically robust than a principal component regression.^39^ In addition, the multicollinearity problem, which occurs when an independent variable is highly correlated with one or more other independent variables, can result in overfitting in general multiple regression models. However, the PLS regression method can effectively deal with the multicollinearity problem and therefore it is particularly suitable for our case where some of the characteristics are to some extent interrelated. While PLS regression was first applied in the social sciences, its usefulness in geosciences has been recently proved in many studies.^40–43^

The statistical significancy of the PLS regression analysis is shown using the cross-validated *R*^2^, which is indicating the square of the correlation between the actual and predicted values and it is called *Q*^2^ in the PLS analysis. A PLS analysis is assumed to be statistically significant if the *Q*^2^ value is greater than or equal to 0.0975.^38^ The PLS analysis can also give the percentage of variance of the dependent variable explained by the PLS components.^44^ In addition, the PLS analysis provides an estimate of the importance of each of the independent variables. This is called the variable importance in projection (VIP) score. The VIP score represents the statistical contribution of each independent variable to the overall fitted PLS regression across all latent vectors.^45^ The higher the VIP score is, the higher is the importance of that independent variable in explaining the variance of the dependent variable.^46^

The normalized PM_2.5_ concentration was used as the dependent variable in the PLS regression, whereas *z*_0_/*z*_H_, *f*^50^_trees_, *D*_road_ and *λ*_S_ were used as the explanatory variables. The PLS analysis was performed to examine the statistical significance of the effect of urban morphological characteristics on the normalized PM_2.5_ concentrations. In addition, the variance explained by the urban morphological characteristics was examined and the importance of the individual characteristics was identified.

### 2.4 Description of the urban morphological characteristics used in the study

**Fig. 2:**
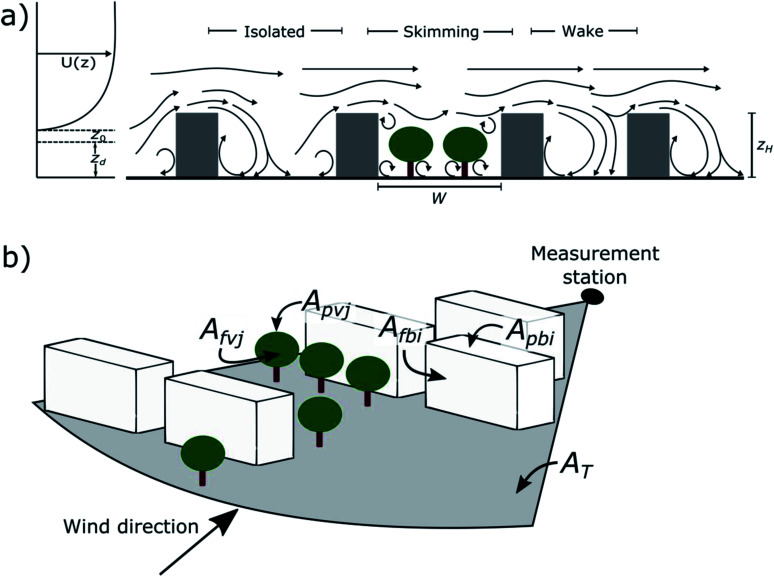
Schematic illustration of (a) street canyon ventilation and (b) the urban surfaces used in the morphometric analyses. *U*(*z*) is the mean wind velocity as a function of height, *z*_0_ is roughness length, *z*_d_ is zero-plane displacement height, *W* is street canyon width, *z*_H_ is mean building height, *A*_T_ is the total surface area, *A*_fv*j*_ and *A*_fb*i*_ are frontal area of individual tree or building, respectively, and *A*_pv*j*_ and *A*_pb*i*_ are plan area of individual tree or building, respectively. The figures are not in scale and the roughness elements relevant to airflow are shown as generic obstacles.

It is challenging to estimate the wind velocity within the roughness sublayer, but the profile above the surface obstacles is generally well approximated by a logarithmic wind profile with the addition of a displacement height.^47^ The roughness length is representing the height above the zero-plane displacement (*z*_d_) where the theoretical logarithmic wind profile is going to zero (Fig. 2a and Table 1).

**Table 1:** Notations used in the study. The variables are calculated as a function of the indented variables below them

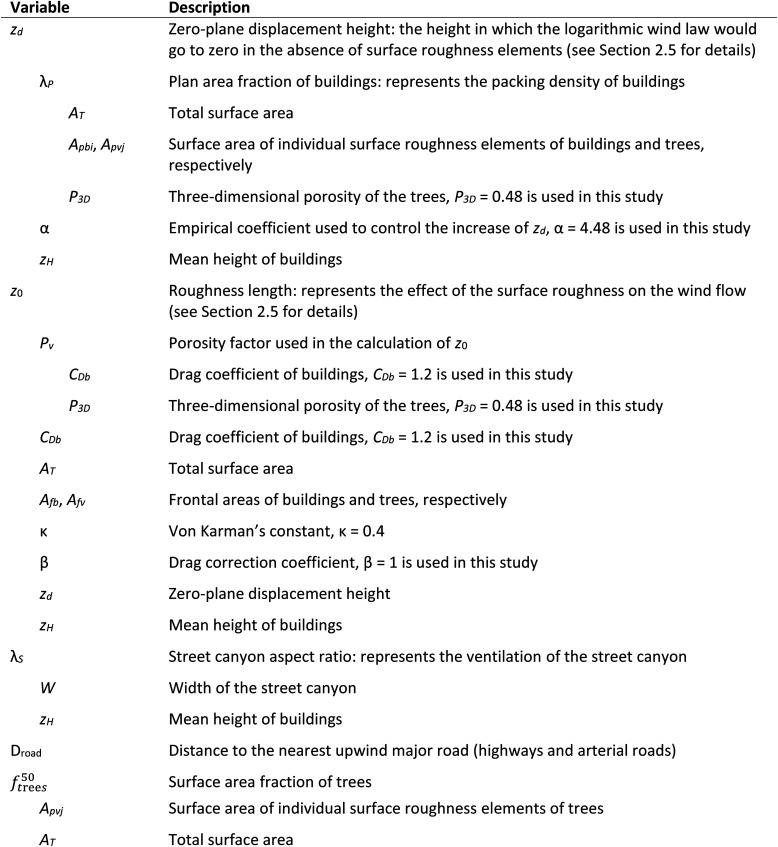

Urban trees can decrease the ventilation of street canyons by preventing the wind from above rooftops to penetrate into near-surface levels (Fig. 2a). Thus, the pollutants are not effectively dispersed from street canyons to upper layers, and this causes accumulation of pollutants at the pedestrian level. The fraction of trees was calculated as Σ*A*_pv*j*_/*A*_T_, where *A*_pv*j*_ is the plan area of individual trees and *A*_T_ is the total surface area (Fig. 2b). Similarly, the plan area fraction of buildings (*λ*_P_) used in the calculation of the aerodynamic parameters (see Section 2.5) was calculated as Σ*A*_pb*i*_/*A*_T_, where *A*_pv*i*_ is the plan area of individual buildings (Fig. 2b).

The street canyon aspect ratio is calculated as *z*_H_/*W*, where *z*_H_ is the mean building height and *W* is the street canyon width (Fig. 2a). With *λ*_S_ < 0.3 the street canyon wind is isolated and fairly well ventilated, while with approximately 0.3 < *λ*_S_ < 1.0 there can be a wake effect flow and with approximately *λ*_S_ > 0.65 there can be skimming flow^48–50^ (Fig. 2a) so that the ventilation of the street canyon is reduced. The free surface in the street canyon can be reduced also by trees, causing a wake effect or even skimming flow (Fig. 2a). The street canyon aspect ratio does not change with different wind directions, and therefore the same value of *λ*_S_ was used for all the wind sectors.

The distance to major roads was calculated as the distance from the measurement station to the nearest upwind highway or arterial road.

### 2.5 Urban aerodynamic parameters

The urban aerodynamic parameters, *z*_d_ and *z*_0_, were determined using the morphometric method by MacDonald *et al.*^51^ modified to include trees by Kent *et al.*^52^ In this approach, the vegetation is included in *λ*_P_ using a porosity factor. By including buildings and trees, *λ*_P_ becomes then:1
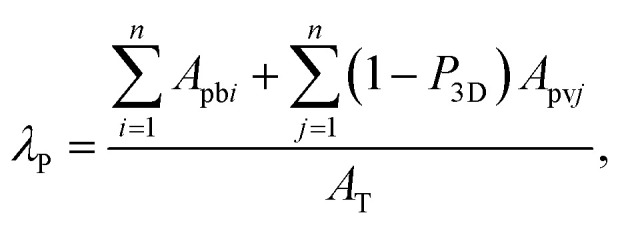
where *i* or *j* refers to each individual built or vegetated roughness element, respectively. *P*_3D_ is the three-dimensional porosity. The recommended values of *P*_3D_ for leaf-on and leaf-off periods are 0.2 and 0.6, respectively.^52^ Although the study period was during the leaf-off period, the value of *P*_3D_ = 0.48 was used because 30% of the urban trees in Nanjing are evergreen broadleaf trees.^53^ The height normalized zero-displacement height is calculated as:^51,52^2
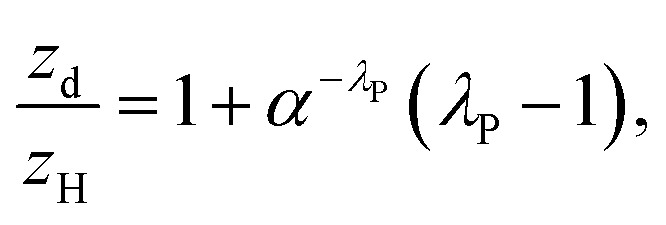
where the constant, *α*, is used to control the increase of *z*_d_ with *λ*_P_.

The porosity factor (*P*_v_) used in the roughness length calculation is^52^ given by:3
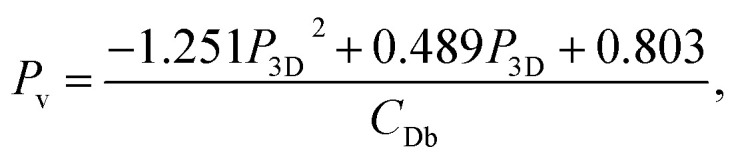
where *C*_Db_ is the drag coefficient for buildings. The height normalized roughness length then becomes:^52^4
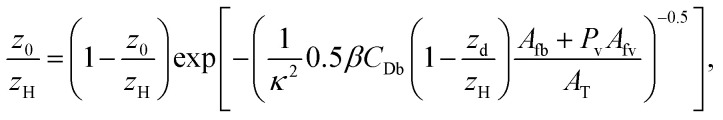
where *κ* is von Karman's constant = 0.4,^54^*β* is the drag correction coefficient and *A*_fb_ and *A*_fv_ are the frontal areas of buildings and trees, respectively. The values of *α* = 4.43, *C*_Db_ = 1.2 and *β* = 1, recommended for staggered arrays,^51^ were used in this study.

**Fig. 3:**
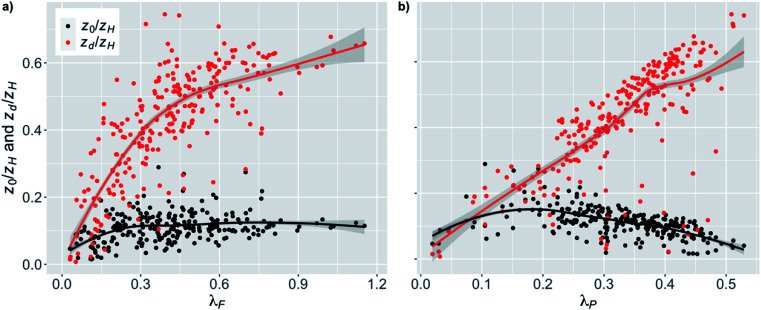
The relation between the height normalized zero-plane displacement (*z*_d_/*z*_H_) and roughness length (*z*_0_/*z*_H_) and the packing density of roughness elements using (a) the frontal area index (*λ*_F_) and (b) plan area fraction (*λ*_P_) with a LOESS^55^ fit line.

This study included 31 observation points with a good spatial coverage and all of them were divided into eight different wind sectors with individually identified urban morphological characteristics. Therefore, there were highly varying conditions examined and the different urban densities were well covered in the study (Fig. 3). Thus, the effect of urban morphological characteristics on near-surface PM_2.5_ concentrations could be examined throughout the whole scale of urban densities typically found in cities.^49^

## 3. Results

### 3.1 Effect of urban morphological characteristics

#### 3.1.1 Pre-COVID period

##### Linear regression analysis for the street canyons and open areas

**Fig. 4:**
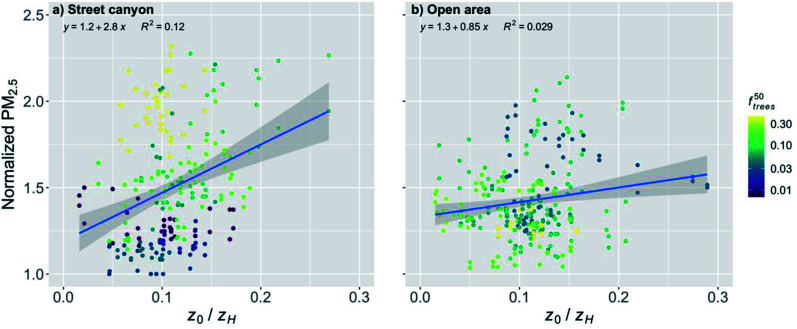
Monthly medians of normalized PM_2.5_ concentrations for stations in (a) street canyons and (b) open areas against the height normalized roughness length (*z*_0_/*z*_H_) during the pre-COVID period. The coloring shows the fraction of trees (*f*^50^_trees_) within 50 m around the station. The shaded area is showing the 95% confidence boundaries.

The roughness length is assumed to be one of the most important urban characteristics in terms of the street canyon ventilation. In this study we examined the effect of the height-normalized roughness length on the near-surface PM_2.5_ concentrations normalized with the background concentration. When the effect of *z*_0_/*z*_H_ on normalized PM_2.5_ concentrations was examined for the stations in street canyons, there was a significant (*p*-value < 0.001) increase with increasing *z*_0_/*z*_H_ (Fig. 4a and Table 2). An increase in *z*_0_/*z*_H_ from 25 percentile to 75 percentile (*i.e.* by the interquartile range, IQR) increased the normalized PM_2.5_ concentration by 9%. However, there was a rather large spread in the scatter (from 1.0 to 2.3 in normalized PM_2.5_). The coloring of the points shows that *f*^50^_trees_ was responsible for much of this spread. The stations with a higher fraction of trees tended to have higher PM_2.5_ concentrations.

**Table 2:** Statistical parameters for fraction of trees within a 50 m radius (*f*^50^_trees_), height-normalized roughness length (*z*_0_/*z*_H_), distance to major roads (*D*_road_) and street canyon aspect ratio (*λ*_S_) against normalized PM_2.5_ concentrations. *R*^2^ is the correlation coefficient for the linear regression, VIP is the variable importance from the partial least squares regression analysis, ΔPM_2.5_ stands for the percentual increase in the normalized PM_2.5_ with an increase of the variable examined by the interquartile range (from 25 percentile to 75 percentile) and the R2Xy is the variance explained by the variables of the partial regression analysis. Statistical significancy of linear regression analyses are shown as: *p*-value <0.001 (***), <0.01 (**), <0.05 (*), <0.1 (.), <1 ( ). See Section 2.3 for statistics explanation

Period	Variable	Street canyons				Open areas			
		*R* ^2^	VIP	ΔPM_2.5_ (%)	R2Xy (%)	*R* ^2^	VIP	ΔPM_2.5_ (%)	R2Xy (%)
Pre-COVID	*f* ^50^_trees_	0.470***	1.68	24		0.084***	1.26	−6	
	*z*_0_/*z*_H_	0.120***	0.85	9		0.029**	0.87	3	
	*D*_road_	0.069***	0.65	−8		0.031**	0.83	−3	
	*λ*_S_	0.003	0.18	—		0.057***	0.99	1	
					59				13
COVID lockdown	*f* ^50^_trees_	0.490***	1.65	22		0.002	—	—	
	*z*_0_/*z*_H_	0.150***	0.91	9		0.026.	—	—	
	*D*_road_	0.076**	0.65	−8		0.012	—	—	
	*λ*_S_	0.005	0.17	—		0.013	—	—	
					73				—

When the stations located in the open areas were examined, the effect of *z*_0_/*z*_H_ was much smaller on the normalized PM_2.5_ concentration than it was for the street canyons (Fig. 4b and Table 2), but it was still significant (*p*-value < 0.01). This was quite expected since the increased surface roughness due to the increased *z*_0_/*z*_H_ in the upwind direction could actually induce more turbulence and therefore even benefit the ventilation of open areas, whereas the increasing *z*_0_/*z*_H_ was clearly deteriorating the PM_2.5_ air quality in the street canyons. In addition, the nearby trees did not seem to have a similar effect as in street canyons. An increase in *z*_0_/*z*_H_ by the IQR increased the normalized PM_2.5_ concentration by 3% in open areas.

**Fig. 5:**
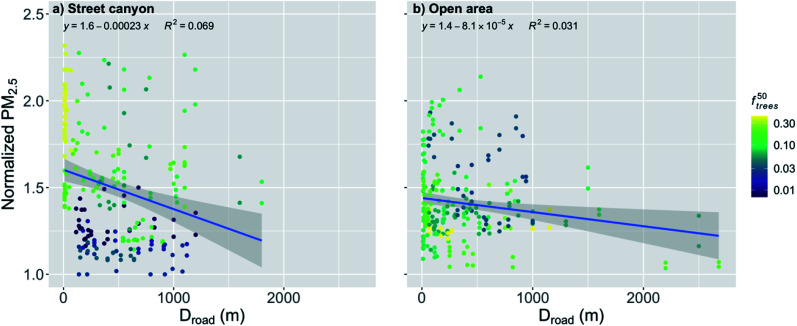
Monthly medians of normalized PM_2.5_ concentrations stations in (a) street canyons and (b) open areas against the distance to major roads (*D*_road_) during the pre-COVID period. The coloring shows the fraction of trees within 50 m radius of the measurement station (*f*^50^_trees_). The shaded area is showing the 95% confidence boundaries.

When the distance to major roads increased, the normalized PM_2.5_ concentration decreased significantly (*p*-value < 0.001) for the stations located in the street canyons (Fig. 5a and Table 2). However, the correlation is quite weak (*R*^2^ = 0.069) due to the high variation. An increase in *D*_road_ by the IQR decreased the normalized PM_2.5_ by 8%. Again, especially close to the major roads, *f*^50^_trees_ seems to play an important role by increasing the concentration with an increasing fraction of trees, which explains much of the rather high variation.

The spread of the scatter is slightly smaller when examining only the stations located in the open areas (Fig. 5b and Table 2), but again the correlation is quite weak (*R*^2^ = 0.031). The other urban morphological characteristics (*e.g.*, *z*_0_/*z*_H_) are assumed to have a smaller effect on the stations in open areas which might partly explain the smaller variation in the open areas. The distance to major roads for the open areas had a significant (*p*-value < 0.001) effect on the normalized PM_2.5_ concentration (Fig. 5b and Table 2), and an increase in *D*_road_ by the IQR decreased the normalized PM_2.5_ by 3%.

**Fig. 6:**
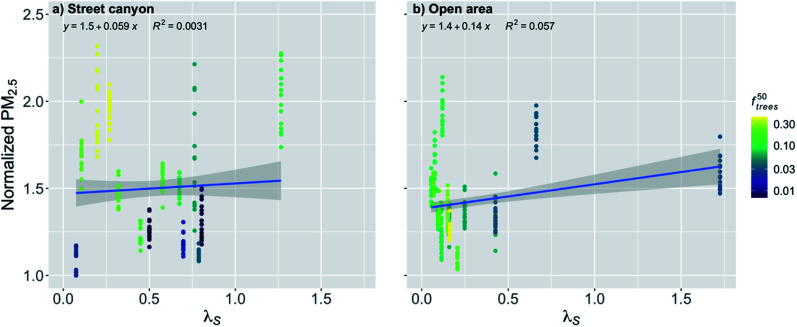
Monthly medians of normalized PM_2.5_ concentrations for stations in (a) street canyons and (b) open areas against the street canyon aspect ratio (*λ*_S_) during the pre-COVID period. The coloring shows the fraction of trees within 50 m radius of the measurement station (*f*^50^_trees_). The shaded area is showing the 95% confidence boundaries.

The effect of street canyon aspect ratio (*λ*_S_) on the normalized PM_2.5_ concentration was nonsignificant (*p*-value > 0.05) for the street canyons (Fig. 6a and Table 2). However, it can be seen that if the street canyon has a large fraction of trees, the concentrations tend to be higher, which is expected, since it can make the free area in the street canyon smaller and therefore decrease the ventilation.

In the open areas, due to the large width of the areas, most of the stations have obviously quite low *λ*_S_, except one station that is next to a very tall building (Fig. 6b). This makes the linear regression fit statistically significant (*p*-value < 0.001), even though there are quite a lot of seemingly random variation between the stations with lower *λ*_S_ in the area of isolated flow. There are only few stations in the area of wake effect or skimming, where the *λ*_S_ is assumed to have an effect on the ventilation conditions, and therefore the fit between the points might not be representative, especially as the *λ*_S_ is assumed to affect more substantially in the street canyons where the linear regression was nonsignificant. When the linear relationship in the open areas is examined, an increase in *λ*_S_ by the IQR increased the normalized PM_2.5_ concentration by 1% (Fig. 6b and Table 2). However, this result should probably be considered doubtful and strong conclusions based on it should be avoided.

**Fig. 7:**
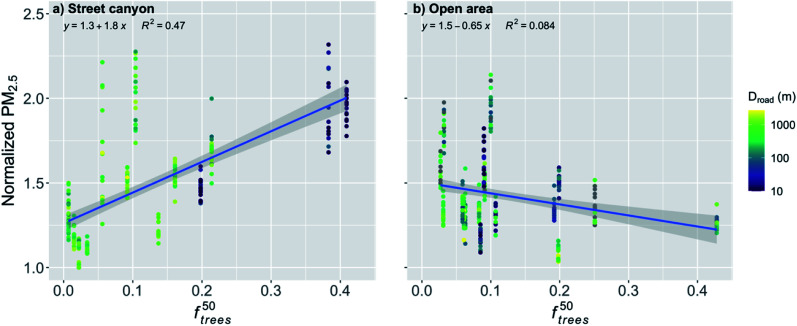
Monthly medians of normalized PM_2.5_ concentrations for stations in (a) street canyons and (b) open areas against the fraction of trees within 50 m radius of the station (*f*^50^_trees_) during the pre-COVID period. The coloring shows the distance to major roads (arterial roads and highways). The shaded area is showing the 95% confidence boundaries.

In order to further analyze the effect of the trees, the normalized PM_2.5_ was plotted against *f*^50^_trees_ (Fig. 7). It is obvious that the trees within a 50 m radius had a significant effect (*p*-value < 0.001) on the normalized PM_2.5_ concentration for the stations in the street canyons (Fig. 7a and Table 2). An increase in *f*^50^_trees_ by the IQR increased the normalized PM_2.5_ concentration by 24%. The distance to major roads is not expected to have a substantial effect on this relationship because the trees around the observation station are likely to accumulate local pollutants and therefore the transport from roads further away should not have a strong effect.

The effect of *f*^50^_trees_ seemed to be reversed for the open areas compared to the street canyons, and the normalized PM_2.5_ concentration was actually decreasing significantly (*p*-value < 0.001) with increasing *f*^50^_trees_ (Fig. 7b and Table 2). An increase in *f*^50^_trees_ by the IQR decreased the normalized PM_2.5_ by 6%. The stations in the open areas are located for example next to a sports field, where local emissions are negligible and therefore there is no substantial accumulation of local pollutants and the purification effect of vegetation is dominating, which is agreeing well with previous studies.^56^

##### Partial least squares (PLS) regression analysis

To further analyze the statistical significance of the urban morphological characteristics and the importance of different characteristics individually on the PM_2.5_ concentration, a partial least squares (PLS) regression analysis was performed (see Section 2.3). In the PLS analysis the quantity *Q*^2^ is used to measure the statistical significancy of the analysis with higher values indicating higher statistical significancy. When using *f*^50^_trees_, *z*_0_/*z*_H_, *D*_road_ and *λ*_S_ as the explanatory variables, the value of *Q*^2^ was 0.4723 for the street canyons. This is an indication of a rather strong statistical significancy, since the limit for statistically significant analysis is generally assumed to be 0.0975.^38^ The variance explained by these urban characteristics based on the PLS analysis was 59%. Therefore, based on these results, the variation of local pollutant emissions and characteristics not covered in this study were responsible only for less than half of the spatial variation within the downtown Nanjing and the urban morphological characteristics studied were very important in determining the spatial variation of PM_2.5_ concentrations. For the open areas, the analysis was also statistically significant (*Q*^2^ = 0.1064). The variance explained for the open areas was 13%, much smaller than for the street canyons.

The VIP score is a measure of the importance of the individual variables in the PLS analysis, where the variables with higher values are assumed to be more important. The VIP scores for different characteristics for the street canyons were 1.68, 0.85, 0.65 and 0.18 for *f*^50^_trees_, *z*_0_/*z*_H_, *D*_road_ and *λ*_S_, respectively (Table 2). For the open areas, the VIP scores were 1.26, 0.87, 0.83 and 0.99 for *f*^50^_trees_, *z*_0_/*z*_H_, *D*_road_ and *λ*_S_, respectively (Table 2). These analyses, in addition to the scatter plots presented before, further support the hypothesis that the *z*_0_/*z*_H_ and especially *f*^50^_trees_ are the two most important urban morphological characteristics dictating the local variation of near-surface PM_2.5_ concentrations in urban areas.

#### 3.1.2 COVID lockdown period

**Fig. 8:**
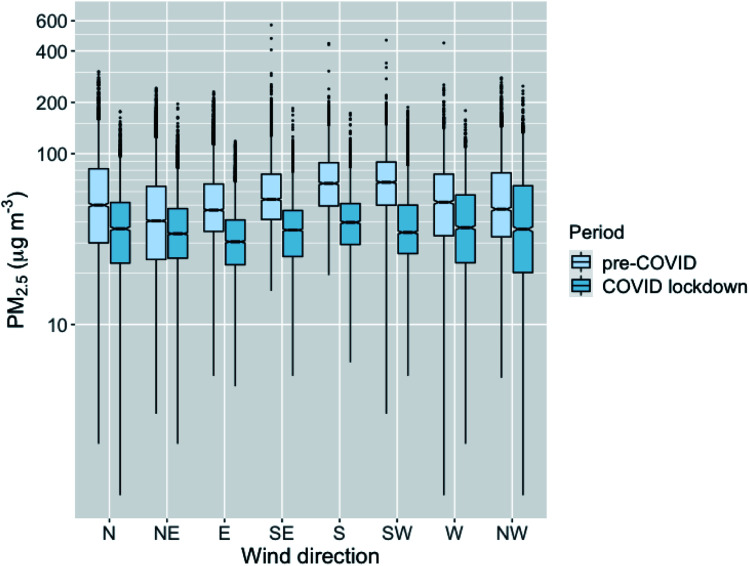
The PM_2.5_ concentrations of all stations in same boxplots for different wind directions and for pre-COVID and COVID lockdown periods. The medians are shown with horizontal line, the notches represent the 95% confidence boundaries, the boxes are showing the interquartile range (IQR) and the whiskers show 1.5 times the IQR.

The COVID lockdown affected the local pollutants substantially in Nanjing, as also reported in numerous other studies.^57–59^ Based on our results, the PM_2.5_ concentration dropped approximately 31% when taking the difference in the medians of the values over all the stations for pre-COVID and COVID lockdown periods. The decrease in PM_2.5_ for different wind directions varied from 16% (NE) to 49% (SW) (Fig. 8). The COVID lockdown mainly affected local pollutant emissions, while the industrial emissions stayed relatively similar.^60^ Therefore, the decrease for the northeast direction was presumably the smallest due to the industrial area located in that wind direction.

The decrease in the pollutants due to the COVID restrictions allowed us to examine the effect of urban morphological characteristics also under a substantially different emission scenario. The relatively larger amount of transported emissions compared to local emissions during the COVID lockdown period should not affect the analyses substantially, since the effect of transported emissions was minimized by the normalization of the PM_2.5_ concentrations using the background concentration (see Section 2.3).

##### Linear regression analysis for the street canyons and open areas

The effect of urban morphological characteristics on the normalized PM_2.5_ concentrations stayed mainly very similar compared to the pre-COVID period, especially in the street canyons (Table 2), even though the pollutant concentrations dropped due to the COVID restrictions. This is presumably because we were focusing on the relative change through the normalized PM_2.5_ concentrations and therefore the changes in pollutant emissions did not have such strong influence on the relationship between the urban morphological characteristics and the spatial variation of normalized PM_2.5_. However, since the pre-COVID analyses are based roughly on monthly medians of two months and the COVID period has only medians for roughly one month (see Section 2.3), the analyses for the two periods might not be directly comparable.

**Fig. 9:**
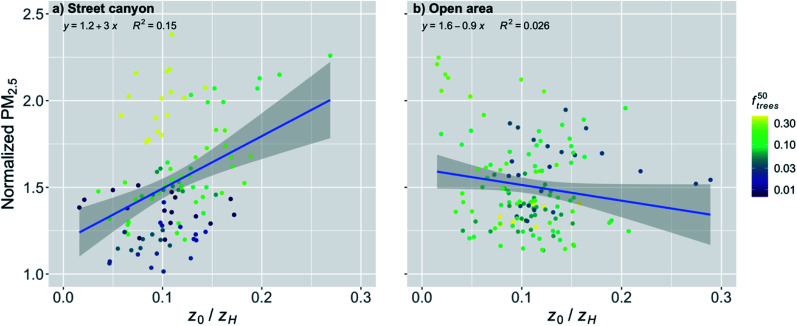
Monthly medians of normalized PM_2.5_ concentrations for stations in (a) street canyons and (b) open areas against the height normalized roughness length (*z*_0_/*z*_H_) during the COVID lockdown period. The coloring shows the fraction of trees (*f*^50^_trees_) within 50 m around the station. The shaded area is showing the 95% confidence boundaries.

The effect of *z*_0_/*z*_H_ on the normalized PM_2.5_ was very similar also during the COVID lockdown period for the stations in street canyons (Fig. 9a and Table 2). An increase in *z*_0_/*z*_H_ by the IQR increased the normalized PM_2.5_ by 9%. Now during the COVID lockdown, the effect of the increased turbulence induced by the roughness elements in the upwind direction can be seen clearly, as the normalized PM_2.5_ decreased with an increasing *z*_0_/*z*_H_ (Fig. 9b and Table 2), which is opposite to the behavior in the street canyons. However, this relationship was statistically nonsignificant (*p*-value = 0.059).

**Fig. 10:**
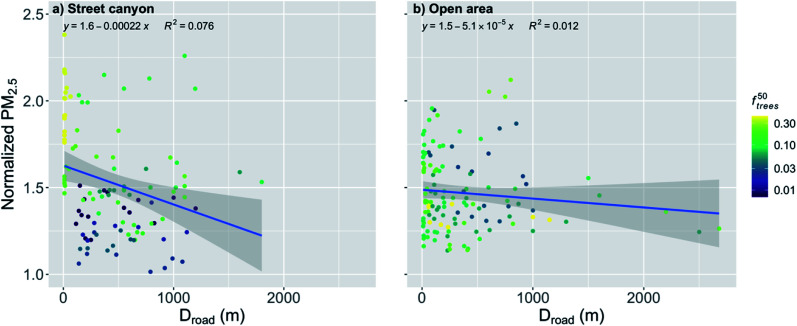
Monthly medians of normalized PM_2.5_ concentrations stations in the (a) street canyons and (b) open areas against the distance to major roads (*D*_road_) during the COVID lockdown period. The coloring shows the fraction of trees within 50 m radius of the measurement station (*f*^50^_trees_). The shaded area is showing the 95% confidence boundaries.

During the COVID lockdown period, an increase in *D*_road_ by the IQR decreased the normalized PM_2.5_ by 8% for the stations in the street canyons (Fig. 10a and Table 2). However, the correlation was quite weak (*R*^2^ = 0.076), even though the relationship was statistically significant (*p*-value < 0.01). The high variation leading to the weak correlation can again be partly explained by the effect of the nearby trees similarly to pre-COVID period. For the stations in the open areas, the relationship was much smaller than in the street canyons and statistically nonsignificant during the COVID lockdown, even though a slight decrease with an increasing *D*_road_ can be seen (Fig. 10b).

**Fig. 11:**
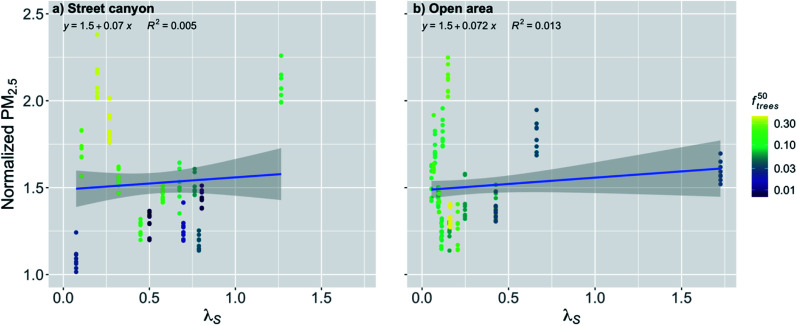
Monthly medians of normalized PM_2.5_ concentrations for stations in (a) street canyons and (b) open areas against the street canyon aspect ratio (*λ*_S_) during the COVID lockdown period. The coloring shows the fraction of trees within 50 m radius of the measurement station (*f*^50^_trees_). The shaded area is showing the 95% confidence boundaries.

The effect of *λ*_S_ during the COVID lockdown period was nonsignificant for the stations in the street canyons and for the open areas (Fig. 11 and Table 2). For the street canyons, the effect of *f*^50^_trees_ could be seen again by inducing higher normalized PM_2.5_ concentrations when there was higher fraction of trees (Fig. 11a).

**Fig. 12:**
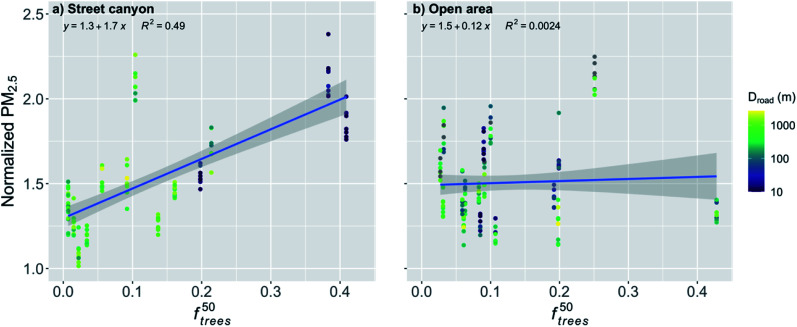
Monthly medians of normalized PM_2.5_ concentrations for stations in (a) street canyons and (b) open areas against the fraction of trees within 50 m radius of the station (*f*^50^_trees_) during the COVID lockdown period. The coloring shows the distance to major roads (arterial roads and highways). The shaded area is showing the 95% confidence boundaries.

For the stations in the street canyon during the COVID lockdown, *f*^50^_trees_ had a significant effect (*p*-value < 0.001) on the normalized PM_2.5_ (Fig. 12a and Table 2). An increase in *f*^50^_trees_ by the IQR increased the normalized PM_2.5_ by 22%. Now, *f*^50^_trees_ in the open areas had a nonsignificant effect on the normalized PM_2.5_ concentration (Fig. 12b and Table 2).

##### Partial least squares (PLS) regression analysis

When examining the statistical significancy of the effect of urban morphological characteristics using the PLS analysis, the results for COVID lockdown period are again rather similar to pre-COVID results. When using *z*_0_/*z*_H_, *f*^50^_trees_, *D*_road_ and *λ*_S_, the value of *Q*^2^ was 0.5488 for street canyons, which indicates strong statistical significancy. For the open areas, the analysis was nonsignificant (*Q*^2^ = 0.0171). The VIP scores for individual characteristics for the street canyons were 1.65, 0.91, 0.65 and 0.17 for *f*^50^_trees_, *z*_0_/*z*_H_, *D*_road_ and *λ*_S_, respectively (Table 2). The relative importance of the individual characteristics during the COVID lockdown period stayed mostly very similar to those during pre-COVID period for the street canyons. The variance explained during the COVID lockdown period by these urban characteristics based on the PLS analysis was 73%. Therefore, similarly to the pre-COVID period, the urban morphological characteristics were the most important factor in explaining the local spatial variation of PM_2.5_ concentrations during the COVID lockdown.

#### 3.1.3 The cleaning effect of trees under different pollution levels

**Fig. 13:**
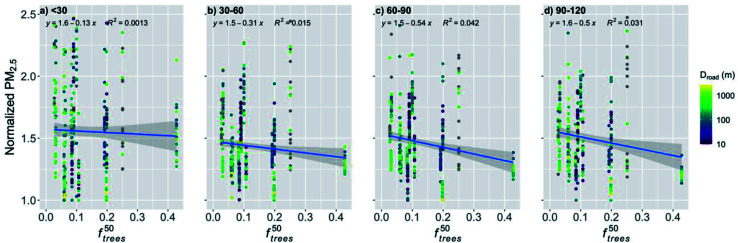
Monthly medians of normalized PM_2.5_ concentrations for station in the street canyons against the fraction of trees within a 50 m radius of the station (*f*^50^_trees_) for the whole study period. The data was stratified into four different PM_2.5_ levels: (a) <30, (b) 30–60, (c) 60–90 and (d) 90–120 μg m^−3^. The coloring shows the distance to major roads (arterial roads and highways). The shaded area is showing the 95% confidence boundaries.

The cleaning effect of nearby trees was significant during the pre-COVID period in the open areas (Fig. 7b), but nonsignificant during the COVID lockdown (Fig. 12b) under substantially lower PM_2.5_ concentrations. Therefore, the cleaning effect of trees under different pollution levels was further examined. We included data for the whole study period and stratified it into four different hourly PM_2.5_ levels (<30, 30–60, 60–90 and 90–120 μg m^−3^; Fig. 13) which were used to calculate the monthly medians. The cleaning effect in the open areas was nonsignificant (*p*-value = 0.197) only with the lowest pollution (<30 μg m^−3^, Fig. 13a). The cleaning effect was significant when the PM_2.5_ concentration exceeded 30 μg m^−3^ (Fig. 13b–d) and it seemed to be more efficient with higher pollution (>60 μg m^−3^, Fig. 13c and d), while staying rather similar with the two highest concentration levels. The *p*-values were better with higher pollutant concentrations (*p*-value ≪ 0.001 and *p*-value = 0.003 for 60–90 and 90–120 μg m^−3^, respectively) when compared to the *p*-value with the PM_2.5_ concentration of 30–60 μg m^−3^ (*p*-value = 0.0124). Similarly, the correlation coefficient (*R*^2^) increased from 0.015 (for 30–60 μg m^−3^, Fig. 13b) to 0.042 and 0.031 (for 60–90 and 90–120 μg m^−3^, respectively; Fig. 13c and d).

## 4. Discussion

In this study, the effect of *z*_0_/*z*_H_ on the near-surface PM_2.5_ concentration was the second most important characteristic studied for both street canyons and open areas during the pre-COVID period and for the street canyons during the COVID lockdown period. In the open areas, *z*_0_/*z*_H_ had the strongest correlation with the normalized PM_2.5_ concentration of the studied characteristics during the COVID lockdown period and an increasing *z*_0_/*z*_H_ seemed to be even benefiting the PM_2.5_ air quality presumably due to the increased turbulence induced by the increased surface roughness in the upwind direction. The roughness length has been identified as one of the most important urban morphological characteristics in terms of air quality also by Edussuriya *et al.*^61^ where the effect of 21 different morphological characteristics were studied. However, only 6 of the variables (complete aspect ratio, occlusivity, roughness height, zero-plane displacement height, total building volume/number of buildings, and standard deviation of height) used by Edussuriya *et al.* were found to vary significantly at district level^62^ and therefore also responsible for the spatial variation of air quality within the city. Many of these variables are the same or similar as used in this study, but for example occlusivity was left out of this study since it requires very detailed data on buildings, which are not often available. The standard deviation of building height has been found to increase the vertical turbulent flux rates^63^ and therefore also to improve the near-surface air quality.^36,63^ However, in this study the standard deviation of the building height was found to have an insignificant effect on PM_2.5_ concentrations in all the scenarios. Based on our results, the height-normalized roughness length was representing better the effect of surface roughness on the PM_2.5_ air quality in this case. Therefore, the standard deviation of the building height was left out of further analyses in this study.

Surprisingly, the street canyon aspect ratio was not a very important factor in determining the near-surface PM_2.5_ air quality, even though its importance to street canyon ventilation has been shown in many wind tunnel and modelling studies.^64,65^ However, these studies have usually focused on idealized street canyons without trees present. The reason for the weak effect might have been that the fraction of trees in the street canyon was affecting the local PM_2.5_ air quality so much that the relationship with *λ*_S_ and normalized PM_2.5_ was not strong, which has also been also found by Gromke and Ruck.^66^ This indicates that the street canyon aspect ratio alone does not represent very well ventilation conditions in real urban environments where trees are commonly present in street canyons.

An exponential relationship of *D*_road_ and many air pollutant concentrations has been suggested by several studies.^36,67^ However, the relationship between the PM_2.5_ concentration and distance to major roads has been shown to be more gradual and closer to a linear relationship,^67,68^ even though some studies have reported an exponential relationships also for PM_2.5_.^69,70^ The assumption of a linear relationship seemed to fit well with our results in the open urban areas (Fig. 5b and 10b), but there was visible fast decay with short distances when the distance from major roads was increasing in the street canyons (Fig. 5a and 10a). However, this steep increase in the normalized PM_2.5_ seemed to be mostly induced by *f*^50^_trees_ in the street canyons, and the earlier studies did not take into account the effect of the nearby trees on the PM_2.5_ concentrations. Therefore, further research on the nature of this relationship in different urban areas would be needed.

We showed that the trees located nearby the observation stations deteriorated significantly the PM_2.5_ air quality in the street canyons. Similar results of the effect of trees on the air quality have been found also in other studies. Di Sabatino *et al.*^28^ found that trees decreased the wind velocities under the tree canopy by up to 79% during the leaf-on period and 39% during the leaf-off period when compared to wind velocities at the roof top, and therefore increased the pollutant concentration by up to 20% in Lecce, Italy. Karttunen *et al.*^30^ found an increase of up to 42% in the PM_2.5_ concentration when compared to a street canyon with no trees in Helsinki, Finland. Jeanjean *et al.*^29^ found that trees trapped air pollution under the canopy and increased the PM_2.5_ concentration locally by approximately 7% in London, UK. Jeanjean *et al.*^29^ concluded that even though there is deposition of PM_2.5_ on the trees, the aerodynamic effect decreasing the ventilation and accumulation of pollutants under the canopy increases the PM_2.5_ concentration four times more than the cleaning effect by deposition decreases the pollutant concentration. In addition, certain tree species can release significant amounts of BVOC emissions.^33^ The emissions of isoprene contribute to the formation of near-surface ozone in urban areas, and monoterpenes and sesquiterpenes can increase PM_2.5_ and PM_10_ concentrations.^71^ Here we showed an increase of 22 and 24% in the normalized PM_2.5_ concentration in the street canyons for the pre-COVID and COVID lockdown periods, respectively, when *f*^50^_trees_ increased by the IQR, which agrees well with the earlier findings and with the larger range given in Abhijith *et al.*^56^

However, we also showed that in the open areas the trees nearby the observation station actually clean the air during the pre-COVID period, and we reported a significant decrease in the normalized PM_2.5_ concentration by 6% with an increase in *f*^50^_trees_ by the IQR. During the COVID lockdown period, the effect of *f*^50^_trees_ was insignificant in open areas. This might be due to the lower pollutant concentrations due to the COVID restrictions, as the pollutant removal by the trees has been shown to be more effective with increasing pollutant concentrations,^72^ which can be seen also in our results (Fig. 13).

Due to the cleaning effect of vegetation, planting of urban trees has often been seen as a solution to improve urban air quality^26,27^ and in urban design the green city idea with a lot of urban trees have been thought to be one with pure air.^73^ However, planting trees in street canyons in order to purify air in urban areas should be carefully reconsidered, since it tends to deteriorate the air quality locally. Especially, the use of urban trees for alleviating a local pollution hotspot should be avoided, since it would most likely just accumulate the emissions from the hotspot and make the local pollution even worse. Instead, the urban planners should include large open areas with a high fraction of vegetation, for example urban parks with trees and low vegetation (*e.g.*, shrubs and grass),^74,75^ as pointed out also by the results of this study. In addition, low vegetation (*e.g.*, hedges), that do not capture the street level pollutants under the canopy,^56^ planted between the road traffic and pedestrian lanes in the street canyons have been shown to improve air quality in the pedestrian lanes and therefore protecting the pedestrians from pollutants coming from the road.^56^

However, urban trees have an important role in controlling the human thermal comfort in cities,^76,77^ that suffer from high temperatures during the summertime as in Nanjing. In addition, urban trees also improve the well-being of the citizens.^78^ Therefore, some number of trees should be planted also in the street canyons to protect the pedestrians from the heat and to make the urban areas more pleasant for the citizens. In order to minimize the deterioration effect on air quality, special attention and further research should be given to the choice of tree species, tree placement and tree heights. For example, variation in the tree height has been shown to be effective in increasing turbulence, compared to homogenous canopy height, and therefore leading to a smaller deterioration of the ventilation and air quality at the pedestrian level.^30^ In addition, it is important to understand the effect of trees on the spatial variation of pollutant accumulation/removal and on the spatial variation of BVOC emissions, in order to understand better the spatial variation of new particle formation in cities.^1^

The correlations in the linear regression analyses were rather weak even for the statistically significant cases (from 0.029 to 0.49, Table 2). Therefore, we performed also the PLS analysis which is a statistically more robust method for multi-parameter problems, especially those with interrelated variables (see Section 2.3). The variable importance obtained from the PLS analysis was giving very similar results to the values from the regression analyses further supporting the results (Table 2). The variance of normalized PM_2.5_ explained in the street canyons during the COVID lockdown period by the urban characteristics studied based on the PLS analysis was 73%, which was much higher than the variance explained during the pre-COVID period (59%). This might be explained by the reduced emissions from the local point emission sources due to the restrictions during the COVID lockdown, thereby giving more importance to the morphological characteristics in the spatial variation. In addition, due to the reduced emissions, the accumulation and dispersion of pollutants were also reduced. Therefore, presumably the linear regressions and the PLS analysis in open areas during the COVID lockdown were statistically nonsignificant for all the variables studied. However, in both of these scenarios, the urban morphological characteristics studied were the most important factors in explaining the local spatial variation of PM_2.5_ concentrations in street canyons.

For the open areas, the effects of some characteristics were reversed compared to the street canyons, for example nearby trees seemed to improve the PM_2.5_ air quality. This should be taken carefully into account in urban planning. In addition, the variance explained by the characteristics in the open areas was much smaller than for the street canyons, and therefore further studies would be required in order to understand better the factors affecting the air quality in open areas. Also, the seasonal variation in the effect of the urban morphological characteristics on the PM_2.5_ air quality should be considered in future studies. The normalization of the PM_2.5_ concentrations used in this study should minimize the effect of varying meteorological conditions, but for example the variating leaf area index of the vegetation is assumed to affect the accumulation of pollutants and the cleaning effect of the trees.

## 5. Conclusions

In this study we examined the effect of urban morphological characteristics on the spatial variation of near-surface normalized PM_2.5_ concentrations. We focused on the most commonly used urban morphological characteristics that are rather easily defined from commonly available datasets. The study included 31 measurement stations in the Nanjing downtown area located in street canyons and open areas, covering the whole scale of urban densities typically found in cities.

The effect of nearby trees was identified to be the most important urban morphological characteristic defining the near-surface pollutant concentrations and the height-normalized roughness length as the second most important. The street canyon aspect ratio was not representing well the near-surface PM_2.5_ air quality, since the fraction of trees within the street canyons had such a strong influence on the normalized PM_2.5_ concentrations. The variance explained by the urban morphological characteristics studied explained 59 and 73% of the variance of the normalized PM_2.5_ concentrations in the street canyons during the pre-COVID and COVID lockdown periods, respectively, which indicates that the characteristics studied were mainly responsible for the spatial variation of the PM_2.5_ air quality in the street canyons of downtown Nanjing.

Since the effect of nearby trees was so dominant compared to other urban morphological characteristics, it emphasizes that the inclusion of the trees in any type of urban planning or urban modelling related to air quality is crucial in order to obtain representative results. In addition, due to the effect of the nearby trees on the local scale air quality, planting of trees in street canyons in order to purify air in urban areas should be carefully reconsidered, since it tends to deteriorate the air quality locally. Instead, the urban planners should prefer large open areas with a high fraction of vegetation, since based on our results, the trees in open areas improve the air quality. However, several studies have shown the competing positive health impact of trees in street canyons as they protect pedestrians from heat in summertime. Therefore, further research and special attention on tree species, tree placement and heights are recommended in order to minimize the negative effects of trees on air quality.

The results obtained in this study can help urban planners to identify the structures most harmful for local PM_2.5_ air quality. In addition, the results can help researchers to evaluate the representativeness of an individual observation station compared to the other parts of the city and when planning a placement of a new measurement station in order to capture the variating conditions in the cities.

The effect of urban morphological characteristics on normalized PM_2.5_ for the street canyons stayed very similar during the COVID lockdown period, even though the pollutant emissions decreased. This indicates that the results obtained in this paper to evaluate the effect of urban morphological characteristics and the identification of the most important individual characteristics could be presumably transferred also to other cities even with different emission scenarios especially for the street canyons.

## Author contributions

TVK and YX conceptualised the study. YX, LJ, DZ and WN were responsible for the data curation. TVK performed the analyses. BW and WQ were responsible for the observations. JS, MK and AJ supervised the study. TVK, YX, PP, SG, VMK and TP wrote the paper. All authors edited and reviewed the article.

## Conflicts of interest

There are no conflicts of interest to declare.

## Supplementary Material
